# Vulvar dermatofibrosarcoma protuberans in a 55-year-old female: A case report, surgical reconstruction approach and literature review

**DOI:** 10.1016/j.gore.2026.102024

**Published:** 2026-01-06

**Authors:** Amanda Detrés, Itzamar Pastrana, Marjolaine Suárez, Ricardo Gómez

**Affiliations:** a Centro Médico Episcopal San Lucas, Department of Obstetrics & Gynecology, Ponce, Puerto Rico; b Centro Médico Episcopal San Lucas, Department of Graduate Medical Education Research Fellow, Puerto Rico

**Keywords:** Vulvar sarcoma, Dermatofibrosarcoma protuberans, Vulvar neoplasms, Soft tissue sarcoma, Vulvar reconstruction, Radical hemivulvectomy

## Abstract

**Background:**

Dermatofibrosarcoma protuberans (DFSP) is a rare, locally aggressive soft tissue sarcoma characterized by a high risk of local recurrence. Vulvar involvement is exceedingly uncommon and often misdiagnosed as a bening lesion, resulting in delayed diagnosis. Complete surgical excision with negative margins is essential but can be challenging in anatomically sensitive areas as the vulva.

**Case presentation:**

A 55-year-old Puerto Rican woman presented with a slowly enlarging lesion of the left labia majora that was initially managed as a furuncle. Progressive growth and symptoms prompted biopsy, which confirmed DFSP. Staging imaging revealed no metastatic disease. The patient underwent a single-stage left radical hemivulvectomy with immedate reconstruction using a V-Y fasciocutaneous advancement flap. Histopathologic examination demonstrated classic DFSP with uniform spindle cellls in a storiform pattern, stong diffuse CD34 positivity, and negative surgical margins. Postoperative recovery was uncomplicated, with excellent wound healing and flap viability.

**Conclusion:**

This case emphasizes the importance of early biopsy of persistent vulvar lesions and highlights the role of multidiciplinary surgical planning. Single-stage radical excision with immediate reconstruction can achieve favorable oncologic and functional outcomes in vulvar DFSP. Long-term surveillance remains essential due to the potential for late local recurrence.

## Highlights

• Vulvar DFSP is a rare malignancy and may be misdiagnosed as a bening vulvar lesion.
• Early biopsy of persistent vulvar masses enables timely diagnosis and limits local tumor progression.
• Histology and CD34 immunopositivity are key to distinguishing DFSP from bening vulvar mimics.
• Margin-negative surgical excision is critical to minimize the high risk of local recurrence.
• Single-stage radical excision with immediate V-Y flap reconstruction can achieve optimal oncologic and functional outcomes.

## 1 Introduction

Dermatofibrosarcoma protuberans (DFSP) is an uncommon soft tissue sarcoma of fibroblastic origin arising in the dermis with subcutaneous infiltration (Xie and Shen, 2025). Its incidence rate is estimated at 6.25 cases per million person-years (Maghfour et al., 2024). Although DFSP may occur anywhere, 50–60% of cases arise on the trunk and extremities (Sinovich et al., 2025). Vulvar involvement is exceedingly rare, with fewer than 100 cases reported and none previously documented in a Hispanic patient (Xie and Shen, 2025). Primary vulvar sarcomas represent only 1.5–5% of all vulvar malignancies (Xie and Shen, 2025). Although metastasis is rare (2%–5%) there is a high potential for local recurrence after resection, exceeding 20% (Maghfour et al., 2024, Darraz et al., 2024).

DFSP predominantly affects adult women, with a mean age of 44.3 years (Xie and Shen, 2025). Owing to rarity and slow progression, vulvar DFSP is frequently mistaken for benign lesions, delaying management. DFSP typically presents as an asymptomatic firm plaque or nodular lesion that may be violaceous to red-brown color, most commonly on the labia majora (Mancari et al., 2024). Ulceration and nodule formation may appear with progression (Xie and Shen, 2025). Common misdiagnoses include bartholin cyst, sebaceous cyst, abscess, dermatofibroma, lipoma among others (Xie and Shen, 2025 Jul 25, Mancari et al., 2024 Jan 3, Nguyen et al., 2017 Jun).

Diagnosis requires a core needle or excisional biopsy (Mancari et al., 2024). Microscopically, demonstrates densely packed spindle cells in a storiform pattern infiltrating the dermis and subcutis, usually with low atypia and low mitotic activity (Xie and Shen, 2025 Jul 25, Sinovich et al., 2025). The tumor cells are arranged within collagenous or myxoid stroma (Hodgson and Dickson, 2023). Immunohistochemically, DFSP shows a strong CD34 and vimentin positivity and negative for factor XIIIIa, S-100 protein, desmin and smooth muscle actin, aiding distinction from mimics (Xie and Shen, 2025). Over 90% of DFSP cases harbor a t(17;22) resulting in COL1A1-PDGFB fusion, promoting tumorigenesis via platelet-derived growth factor signaling (Hao et al., 2020).

The standard treatment for DFSP is surgical resection with the goal of complete removal and clear margins. Margin-negative excision is critical, as inadequate resection is associated with markedly elevated recurrence rates 20–49% (Xie and Shen, 2025). Adjuvant therapies such as radiation are generally reserved for unresectable, recurrent, or metastatic cases.

We report a case of a 55-year-old woman patient with vulvar DFSP managed with single-stage radical hemivulvectomy and immediate V-Y flap reconstruction. This case underscores the need for early biopsy of persistent vulvar lesions and highlights the value of coordinated oncologic and reconstructive planning.

## 2 Case presentation

A 55-year-old Puerto Rican woman with hypertension and class I obesity (BMI 31.4 kg/m^2) presented to her primary care physician in June 2024 with a small, painless lesion on the left labia majora. The lesion was diagnosed as a furuncle and managed conservatively. Over the following months, the patient noted gradual enlargement.

**Fig. 1:**
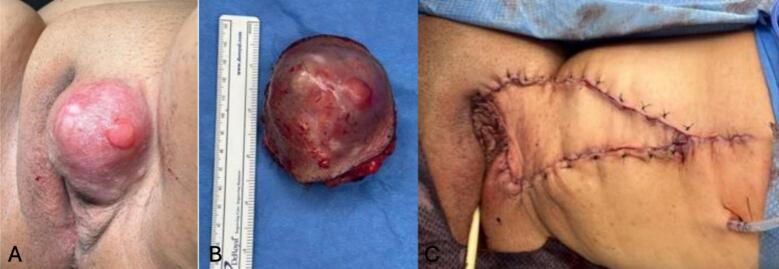
(a) Firm, nodular, protuberant vulvar mass on left labia majora with irregular surface, and with associated skin changes. (b) Vulvar mass after resection, 5 x 4.9 x 4.8 cm. (c) Vulvar reconstruction using a V-Y advancement fasciocutaneous flap technique from the left medial thigh.

By January 2025, the lesion had increased to approximately 4 cm and caused intermittent burning. Examination revealed a firm, nodular, protuberant mass on the left labia majora, with an irregular surface and overlying skin discoloration (Fig. 1). Biopsy of the lesion confirmed DFSP. The patient was subsequently referred to Gynecologic Oncology. A PET/CT scan demonstrated no evidence of distant metastasis.

Following preoperative evaluation and outpatient consultation with Plastic Surgery, a multidisciplinary surgical plan was established. In July 2025, the patient underwent left radical hemivulvectomy. Intraoperatively, a 5 x 4.9 x 4.8 cm brown–red tumor was excised, leaving a 10x3 cm defect. Immediate vulvar reconstruction was performed by a plastic surgeon using a V-Y advancement fasciocutaneous flap harvested from the left medial thigh (16 x 8.5 cm).

**Fig. 2:**
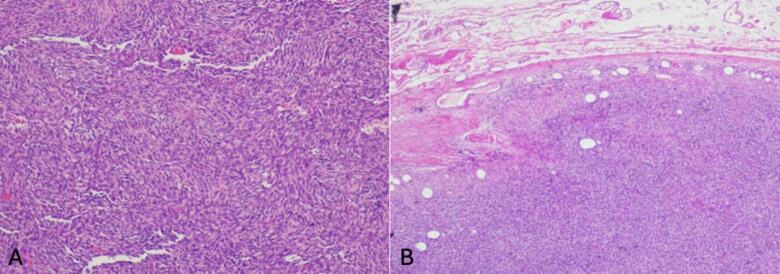
Histopathologic features of vulvar dermatofibrosarcoma protuberans. (a) Shows a poorly circumscribed spindle-cell tumor infiltrating the subcutaneous fat in a honeycomb pattern. (b) Reveals uniform spindle cells in a storiform arrangement with minimal atypia and low mitotic activity, characteristic of DFSP.

Gross pathology of the vulvectomy revealed a 9 x 8 x 5.5 cm skin/subcutaneous specimen containing a 5.1 x 5 cm nodular mass. Microscopically, the tumor showed uniform spindle cells arranged in a storiform pattern, infiltrating the dermis and subcutaneous fat (Fig. 2). No fibrosarcomatous transformation was present. Immunohistochemistry revealed CD34 staining strong and diffuse. All surgical margins were negative, with the closest deep margin measuring 0.3 cm.

Postoperative recovery was uncomplicated, flap viability remained excellent, and early follow-up demonstrated good healing. Long-term surveillance is planned due to risk of local recurrence.

## 3 Discussion

Vulvar Dermatofibrosarcoma protuberans is exceedingly rare, with fewer than 100 cases reported in the literature worldwide in 2025 (Xie and Shen, 2025). Our patient’s profile is consistent with prior cases as vulvar DFSP tends to affect middle-aged women (median age ∼ 46 years) (Mancari et al., 2024). The tumor in our case reached ∼ 5 cm in size, which aligns with the mean lesion diameter (5.3 cm) reported in a recent systematic review (Mancari et al., 2024). This case appears to be the first described in a Hispanic female. The lesion’s indolent growth and benign appearance led to an initial misdiagnosis (as a “furuncle”), because of its rarity, awareness is limited, and diagnosis is frequently delayed or mistaken for benign lesions. Diagnostic delays often exceed 18–24 months (Mancari et al., 2024). In contrast, our patient was diagnosed approximately 12 months after initial lesion onset. This relatively shorter interval to diagnosis suggests that heightened clinical suspicion and early biopsy can lead to more timely management reducing extensive local spread.

Histopathologically, our case demonstrated the classic features of DFSP which are spindle-cell proliferation in a storiform pattern, with infiltration into fat in a “honeycomb” pattern. Immunohistochemistry showed diffuse, strong CD34 positivity, which is a hallmark of DFSP and helps distinguish it from histologic mimics (Neff et al., 2019). The fibrosarcomatous (DFSP-FS) variant portents a more aggressive course with higher metastatic potential and increase to distant spread (most often to lungs), its absence in our patient’s tumor is prognostically favorable (Mancari et al., 2024).

Complete surgical excision with clear margins remains the cornerstone of treatment for vulvar DFSP. Given the tumor’s infiltrative nature beyond grossly visible boundaries, achieving negative margins is paramount to reduce recurrence. Our patient underwent a radical hemivulvectomy with a planned wide margin, and final pathology confirmed margin-negative resection (closest margin 3 mm). Most published cases have been managed with wide local excision (WLE) or partial/radical vulvectomy typically aiming for 2–3 cm gross margins around the tumor (Bertolli et al., 2014). In a largest single-institution series (13 vulvar DFSP cases), all six patients whose initial excisions had positive margins develop local recurrence, whereas only one of seven with clear margins recurred (and that case had a fibrosarcomatous DFSP) (Bertolli et al., 2014). In anatomically sensitive areas like the vulva, however, such wide margins may be challenging without causing significant defects. This has prompted exploration of specialized surgical techniques for margin assessment. To improve margin control, some authors have advocated for mohs micrographic surgery (MMS), even for vulvar lesions (Mancari et al., 2024).

Our case differs from other reports as we performed a single-stage radical hemivulvectomy with immediate reconstruction using a V-Y fasciocutaneous flap from the left medial thigh of the patient performed by a plastic surgeon, rather than staged excision or delayed closure. The post-excisional defect (∼10 x 3 cm) was successfully covered, and the patient healed without complications. In contrast to several reports where reconstruction was delayed pending margin clearance or tissue expansion, our patient underwent single-stage oncologic excision with immediate V-Y flap reconstruction, achieving negative margins without re-excision and rapid full recovery (Bertolli et al., 2014).

Finally, because DFSP is slow-growing and recur locally even many years after excision, long-term surveillance is essential, especially in the vulvar region where recurrences can have profound functional and quality-of-life implications. In the present case, the absence of a high-grade component and negative margins confer a favorable prognosis. At five months postoperatively, the patient remains disease-free. At her follow-up visit in the gynecologic oncology clinic on December 2025, physical examination demonstrated excellent flap uptake with appropriate healing, and no flap-related or postoperative complications have occured to date. Long-term surveillance is crucial, because DFSP can recur locally even after several years of apparent remission (Mancari et al., 2024). Indeed, late recurrences (5–10 years) have been documented, so extended follow-up is recommended.

This case reinforces that with early biopsy, appropriate surgical management, and reconstructive planning, even a rare and locally aggressive tumor like vulvar DFSP can be successfully treated with an excellent outcome involving a multidisciplinary team to manage both the oncologic and reconstructive challenges for the benefit of the patient.

Patient consent

Patient consent is available.

Written informed consent was obtained from the patient for publication of this case report and accompanying images.

## CRediT authorship contribution statement

**Amanda Detrés:** Writing – review & editing, Visualization, Validation, Conceptualization. **Itzamar Pastrana:** Writing – review & editing, Writing – original draft, Data curation, Conceptualization. **Marjolaine Suárez:** Validation, Supervision, Resources, Project administration, Methodology, Conceptualization. **Ricardo Gómez:** Supervision, Resources, Investigation, Conceptualization.

## Declaration of Competing Interest

The authors declare that they have no known competing financial interests or personal relationships that could have appeared to influence the work reported in this paper.
